# Clinical efficacy of integrated traditional Chinese and Western medicine in patients with coronary artery disease

**DOI:** 10.1097/MD.0000000000050810

**Published:** 2026-09-25

**Authors:** Ziyi Chai, Huaying Huo

**Affiliations:** a The First Clinical College of Shanxi University of Chinese Medicine, The Affiliated Hospital of Shanxi University of Chinese Medicine, Jinzhong, Shanxi, China; b Department of Traditional Chinese Medicine, Shanxi Provincial People’s Hospital, Taiyuan, Shanxi, China.

**Keywords:** clinical efficacy, coagulation function, coronary artery disease, lipid metabolism, safety

## Abstract

Coronary artery disease (CAD) remains a major cause of morbidity and mortality worldwide. Although guideline-directed Western medical therapy improves cardiovascular outcomes, residual symptoms remain common. Integrated traditional Chinese and Western medicine is frequently used as an adjunctive strategy in China, but its real-world, short-term clinical associations require further characterization. This retrospective observational study included 215 patients with CAD treated between January 2022 and December 2024, including 106 patients receiving conventional Western medical therapy alone and 109 receiving adjunctive traditional Chinese medicine based on syndrome differentiation. Symptom status, laboratory parameters, overall clinical response, and documented adverse events were compared between groups. Because treatment allocation was nonrandom and the analyses were unadjusted for potential confounders, all between-group findings were interpreted as associations. No statistically significant differences were observed in the measured baseline demographic, clinical, or laboratory characteristics between the 2 groups. After treatment, the integrated-treatment group had lower rates of residual chest pain, chest tightness, and fatigue and greater changes in total cholesterol, triglycerides, low-density lipoprotein cholesterol, high-density lipoprotein cholesterol, fibrinogen, and D-dimer. The observed overall clinical response rate was higher in the integrated-treatment group (90.8% vs 76.4%, *P* = .004). Documented adverse-event incidence did not differ significantly between groups. In this retrospective real-world cohort, adjunctive traditional Chinese medicine was associated with greater short-term improvement in selected symptoms and laboratory parameters and a higher observed clinical response rate, without a detectable increase in documented short-term adverse events. These unadjusted observational findings are exploratory, do not establish causal superiority or improved cardiovascular prognosis, and are insufficient by themselves to modify current guideline-directed CAD practice.

## 1. Introduction

Coronary artery disease (CAD) remains a leading cause of morbidity, mortality, and healthcare utilization worldwide despite substantial advances in pharmacotherapy, risk stratification, and coronary revascularization. Contemporary management has shifted from a narrow focus on obstructive epicardial lesions to a broader framework that recognizes CAD as a chronic, multifactorial atherosclerotic disorder involving lipid accumulation, endothelial dysfunction, vascular inflammation, platelet activation, and thrombosis. Accordingly, long-term treatment requires comprehensive secondary prevention together with sustained symptom control and individualized risk-factor management.^[1–3]^

Although guideline-directed Western medical therapy, including antiplatelet agents, statins and other lipid-lowering therapies, anti-ischemic drugs, blood pressure control, glucose management, and revascularization when indicated, has significantly improved prognosis, a considerable proportion of patients continue to experience recurrent angina, chest tightness, reduced exercise tolerance, and residual cardiometabolic risk. This residual burden reflects the complex pathophysiology of CAD and suggests that conventional treatment alone may not fully address all clinically relevant mechanisms, particularly in patients with persistent symptoms or high thrombo-inflammatory activity.^[4,5]^ In East Asian clinical practice, particularly in China, integrated traditional Chinese and Western medicine has been widely used as an adjunctive strategy for patients with CAD. Traditional Chinese medicine (TCM) is commonly applied on the basis of syndrome differentiation and is intended to complement standard cardiovascular therapy through multitarget regulation of symptom burden, vascular homeostasis, lipid metabolism, and inflammatory activity. Recent reviews and evidence syntheses have suggested that adjunctive TCM may improve angina-related symptoms, enhance some laboratory indicators, and provide additional benefit in selected CAD populations, including patients after percutaneous coronary intervention (PCI). However, the quality of available evidence remains heterogeneous, and further real-world clinical studies are still needed to clarify its practical value under routine treatment conditions.^[6–8]^

Against this background, evaluating integrated traditional Chinese and Western medicine in patients with CAD has both clinical and methodological relevance. In addition to symptomatic improvement, changes in lipid-related and coagulation-related parameters may provide useful information regarding the biological response to treatment, because these domains are closely linked to atherosclerotic progression and ischemic risk. Therefore, the present study was conducted to assess the clinical efficacy and safety of integrated traditional Chinese and Western medicine in patients with CAD in a real-world setting and to provide additional evidence for the optimization of comprehensive treatment strategies for this population.

## 2. Methods

### 2.1. Study design

This study was approved by the Ethics Committee of Shanxi Provincial People’s Hospital (No.:2025-SXPPH-097). This retrospective study enrolled patients with CAD who were treated at our institution between January 2022 and December 2024. This study was conducted in accordance with the principles of the Declaration of Helsinki and was approved by the Institutional Medical Ethics Committee of our hospital. Written informed consent was obtained from all participants or their legal guardians prior to treatment. Eligible participants were adults aged 18 years or older with a confirmed diagnosis of CAD based on clinical manifestations, electrocardiographic findings, coronary imaging, and/or coronary angiography, and who had received integrated traditional Chinese and Western medicine treatment during hospitalization or follow-up. Patients were included if they had complete clinical records, treatment data, and outcome assessment information available for analysis. The exclusion criteria were as follows: patients with severe hepatic or renal dysfunction, malignant tumors, acute or chronic infectious diseases, hematologic disorders, severe autoimmune diseases, or other serious systemic illnesses; patients with congenital heart disease, cardiomyopathy, or significant valvular heart disease; patients with concomitant psychiatric disorders or cognitive impairment that could affect treatment adherence or data reliability; patients who were pregnant or lactating; and patients with incomplete medical records or missing key study variables.

### 2.2. Grouping method

Patients were assigned to different treatment groups according to the actual therapeutic regimen documented in the medical records during hospitalization and follow-up. Specifically, patients who received conventional Western medical therapy alone were classified into the Western medicine group, whereas those who received integrated traditional Chinese and Western medicine therapy on the basis of conventional Western medical treatment were classified into the integrated treatment group. Group allocation was determined retrospectively according to real-world clinical treatment records rather than by random assignment. Because treatment allocation reflected routine clinical practice and was not randomized, confounding by indication and other treatment-selection factors could not be eliminated.

### 2.3. Treatment protocols

All patients received guideline-recommended conventional Western medical treatment for CAD according to their clinical condition. Standard pharmacological management primarily included antiplatelet therapy, lipid-lowering therapy, anti-ischemic treatment, blood pressure control, and glycemic control when indicated. Antiplatelet agents mainly included aspirin and/or P2Y12 receptor inhibitors; lipid-lowering therapy was generally based on statins, with dose adjustment according to lipid profile and cardiovascular risk; anti-ischemic therapy included nitrates, β-blockers, and calcium channel blockers when clinically appropriate. In patients with concomitant hypertension or diabetes mellitus, antihypertensive and hypoglycemic agents were administered in accordance with current clinical guidelines and individualized treatment targets. For patients who underwent PCI, routine post-PCI medical management was also provided, including dual antiplatelet therapy, statin therapy, and other secondary prevention measures as appropriate.

In the integrated treatment group, TCM intervention was administered based on syndrome differentiation and the treatment principles of TCM. Syndrome classification was determined by qualified TCM physicians according to the patients’ clinical manifestations, tongue appearance, pulse characteristics, and overall symptom patterns. Common syndrome types were identified in accordance with established TCM diagnostic principles for chest pain and coronary heart disease. Chinese patent medicines were prescribed according to syndrome differentiation and the patient’s overall clinical status. When individualized modifications were required, treatment adjustments were made according to the basic therapeutic principles of supplementing deficiency, eliminating excess, promoting blood circulation, removing blood stasis, regulating Qi, and relieving chest obstruction, with appropriate modification based on changes in symptoms and disease status. In patients receiving integrated traditional Chinese and Western medicine therapy, TCM treatment was implemented as an adjunct to conventional Western medical management rather than as a replacement for standard care. The integrated therapeutic strategy was developed to combine the benefits of evidence-based Western medicine for secondary prevention and symptom control with TCM interventions aimed at syndrome regulation and overall clinical improvement. The specific treatment regimen was determined by the attending clinicians based on the patient’s disease severity, clinical manifestations, comorbidities, and treatment tolerance, thereby reflecting routine real-world clinical practice.

### 2.4. Data collection

Baseline demographic, clinical, and laboratory data were retrospectively collected from the hospital electronic medical record system using a standardized data extraction form. General information included age, sex, body mass index, smoking history, alcohol consumption history, and major comorbidities, including hypertension, diabetes mellitus, and hyperlipidemia. Clinical characteristics included the type of CAD, disease duration, New York Heart Association functional class, and whether interventional treatment, particularly PCI, had been performed. Baseline laboratory parameters included total cholesterol (TC), triglycerides (TG), low-density lipoprotein cholesterol (LDL-C), high-density lipoprotein cholesterol (HDL-C), fibrinogen (FIB), D-dimer, and routine blood test indices. All data were extracted and checked by trained investigators to ensure accuracy and completeness before analysis.

Data extraction and outcome assessment were performed by 2 trained investigators using a standardized data extraction form. The extracted data were cross-checked by the 2 investigators, and any discrepancies were resolved through discussion; if consensus could not be reached, a third senior investigator made the final decision. Because information on treatment exposure was documented in the same electronic medical records as the clinical outcomes, blinding of the data abstractors to treatment allocation was not feasible. To minimize potential information and assessment bias, laboratory outcomes were obtained directly from the hospital laboratory information system, and clinical symptoms and other outcomes were extracted according to prespecified definitions based on contemporaneous medical records.

### 2.5. Outcome measures

The outcome measures were evaluated in terms of symptom improvement, changes in laboratory parameters, overall clinical response, and safety. Clinical symptom assessment included changes in chest pain, chest tightness, palpitations, shortness of breath, and fatigue before and after treatment, as well as the frequency and duration of angina episodes and the frequency of nitrate use, as documented in the medical records and follow-up data. Laboratory parameters included lipid indices, namely TC, TG, LDL-C, and HDL-C, as well as coagulation and hematological indices, including FIB, D-dimer, white blood cell count (WBC), red blood cell count (RBC), hemoglobin (Hb) level, and platelet count (PLT).

Baseline laboratory values were defined as the measurements obtained closest to treatment initiation within the predefined baseline assessment window, whereas posttreatment values were defined as the measurements obtained closest to completion of the treatment course within the predefined posttreatment assessment window. When more than one eligible measurement was available within the specified window, the measurement closest to the predefined assessment time point was selected for analysis. Patients without eligible baseline or posttreatment measurements within the specified assessment windows were considered to have missing key outcome data and were excluded from the final analytical cohort. The interval between baseline and posttreatment assessments was recorded to evaluate the consistency of follow-up timing between the 2 treatment groups. Follow-up assessments were performed within the predefined posttreatment assessment window, and no systematic difference in assessment timing was identified between the 2 groups.

Overall clinical response was categorized as markedly effective, effective, or ineffective according to the degree of improvement in clinical symptoms, reduction in angina frequency and nitrate use, and changes in relevant laboratory parameters. Markedly effective was defined as marked relief or near disappearance of major symptoms, with a substantial reduction in angina episodes and nitrate use accompanied by clear improvement in laboratory indices; effective was defined as partial symptom relief, reduced angina frequency and nitrate use, and improvement in laboratory indices; and ineffective was defined as no significant improvement or worsening of symptoms and related indicators. The overall response rate was calculated as the proportion of patients classified as markedly effective or effective. Safety outcomes included treatment-related adverse events and complications, such as gastrointestinal discomfort, allergic reactions, bleeding events, hepatic dysfunction, renal dysfunction, and other adverse events documented during treatment and follow-up.

### 2.6. Statistical analysis

Statistical analyses were performed using IBM SPSS Statistics, version 30.0 (IBM Corp.). Continuous variables were expressed as mean ± standard deviation, and between-group comparisons were conducted using the independent-samples *t* test. Categorical variables were presented as frequencies and percentages, and comparisons between groups were performed using the chi-square test. These analyses were unadjusted, and no propensity-score or multivariable confounder-adjustment methods were applied; therefore, the findings represent unadjusted between-group associations. Patients with incomplete medical records or missing key study variables were excluded during cohort construction according to the eligibility criteria. Therefore, no statistical imputation was performed.

## 3. Results

### 3.1. Comparison of baseline characteristics between the 2 groups

A total of 215 patients were included, with 106 in the control group and 109 in the observation group. No significant differences were observed between the 2 groups in baseline demographic characteristics, including age, sex, body mass index, smoking history, or alcohol consumption history (all *P* > .05). The prevalence of major comorbidities, including hypertension, diabetes mellitus, hyperlipidemia, previous myocardial infarction, and previous stroke, was also similar between the 2 groups (all *P* > .05). In addition, there were no statistically significant differences in clinical characteristics, including the distribution of CAD type, disease duration, New York Heart Association class, or the proportion of patients who underwent PCI (all *P* > .05). Baseline laboratory parameters, including TC, TG, LDL-C, HDL-C, FIB, D-dimer, WBC, RBC, Hb, and PLT, did not differ significantly between the 2 groups (all *P* > .05). However, because treatment allocation was nonrandom and the analyses were unadjusted, the absence of statistically significant differences in measured baseline variables does not exclude residual confounding from measured or unmeasured factors (Table 1).

**Table 1:** Comparison of baseline characteristics between the control group and the observation group.

Variable	Control group (n = 106)	Observation group (n = 109)	Test statistic	*P* value
General demographic characteristics				
Age, yrs	64.8 ± 9.1	65.3 ± 8.7	*t* = −0.41	.681
Male sex, n (%)	68 (64.2)	71 (65.1)	χ^2^ = 0.02	.880
BMI, kg/m^2^	24.7 ± 3.0	24.9 ± 3.2	*t* = −0.47	.637
Smoking history, n (%)	36 (34.0)	39 (35.8)	χ^2^ = 0.08	.780
Alcohol consumption history, n (%)	29 (27.4)	31 (28.4)	χ^2^ = 0.03	.860
Comorbidities				
Hypertension, n (%)	63 (59.4)	68 (62.4)	χ^2^ = 0.20	.657
Diabetes mellitus, n (%)	31 (29.2)	35 (32.1)	χ^2^ = 0.21	.649
Hyperlipidemia, n (%)	47 (44.3)	51 (46.8)	χ^2^ = 0.13	.718
Previous myocardial infarction, n (%)	18 (17.0)	20 (18.3)	χ^2^ = 0.07	.793
Previous stroke, n (%)	14 (13.2)	16 (14.7)	χ^2^ = 0.10	.756
Clinical characteristics				
Type of coronary artery disease, n (%)				
Stable angina pectoris	60 (56.6)	58 (53.2)	χ^2^ = 0.25	.881
Unstable angina pectoris	34 (32.1)	38 (34.9)		
Old myocardial infarction/ischemic cardiomyopathy	12 (11.3)	13 (11.9)		
Disease duration, yrs	5.6 ± 3.1	5.8 ± 3.0	*t* = −0.48	.631
NYHA class, n (%)				
Class I	31 (29.2)	29 (26.6)	χ^2^ = 0.19	.908
Class II	53 (50.0)	57 (52.3)		
Class III	22 (20.8)	23 (21.1)		
Underwent PCI, n (%)	59 (55.7)	64 (58.7)	χ^2^ = 0.20	.651
Baseline laboratory parameters				
TC, mmol/L	4.72 ± 0.91	4.79 ± 0.95	*t* = −0.55	.582
TG, mmol/L	1.83 ± 0.72	1.89 ± 0.76	*t* = −0.59	.553
LDL-C, mmol/L	2.88 ± 0.69	2.93 ± 0.72	*t* = −0.52	.604
HDL-C, mmol/L	1.12 ± 0.24	1.10 ± 0.23	*t* = 0.62	.534
FIB, g/L	3.47 ± 0.81	3.55 ± 0.78	*t* = −0.74	.462
D-dimer, mg/L	0.46 ± 0.21	0.49 ± 0.23	*t* = −1.00	.319
WBC, ×10^9^/L	6.78 ± 1.56	6.91 ± 1.62	*t* = −0.60	.550
RBC, ×10^12^/L	4.46 ± 0.47	4.42 ± 0.49	*t* = 0.61	.542
Hb, g/L	136.8 ± 15.7	134.9 ± 16.1	*t* = 0.88	.382
PLT, ×10^9^/L	218.4 ± 56.3	223.7 ± 58.8	*t* = −0.68	.500

BMI = body mass index, FIB = fibrinogen, Hb = hemoglobin, HDL-C = high-density lipoprotein cholesterol, LDL-C = low-density lipoprotein cholesterol, NYHA = New York Heart Association, PCI = percutaneous coronary intervention, PLT = platelet count, RBC = red blood cell count, TC = total cholesterol, TG = triglycerides, WBC = white blood cell count.

### 3.2. Comparison of major symptom improvement after treatment

Before treatment, the prevalence of chest pain, chest tightness, palpitations, shortness of breath, and fatigue did not differ significantly between the control group and the observation group (all *P* > .05). After treatment, the observation group showed significantly lower rates of residual chest pain (24 [22.0%] vs 39 [36.8%], χ^2^ = 5.66, *P* = .017), chest tightness (22 [20.2%] vs 36 [34.0%], χ^2^ = 5.18, *P* = .023), and fatigue (19 [17.4%] vs 31 [29.2%], χ^2^ = 4.20, *P* = .040) than the control group. Although the proportions of patients with residual palpitations (16 [14.7%] vs 24 [22.6%], χ^2^ = 2.25, *P* = .134) and shortness of breath (14 [12.8%] vs 22 [20.8%], χ^2^ = 2.41, *P* = .121) were also lower in the observation group, the differences did not reach statistical significance (Table 2).

**Table 2:** Comparison of residual symptoms after treatment between the control group and the observation group.

Variable	Control group (n = 106)	Observation group (n = 109)	Test statistic	*P* value
Chest pain, n (%)	39 (36.8)	24 (22.0)	χ^2^ = 5.66	.017
Chest tightness, n (%)	36 (34.0)	22 (20.2)	χ^2^ = 5.18	.023
Palpitations, n (%)	24 (22.6)	16 (14.7)	χ^2^ = 2.25	.134
Shortness of breath, n (%)	22 (20.8)	14 (12.8)	χ^2^ = 2.41	.121
Fatigue, n (%)	31 (29.2)	19 (17.4)	χ^2^ = 4.20	.040

### 3.3. Changes in laboratory parameters after treatment

After treatment, greater changes were observed in the observation group than in the control group in several lipid and coagulation parameters. Specifically, the reductions in TC, TG, LDL-C, FIB, and D-dimer, as well as the increase in HDL-C, were all significantly greater in the observation group than in the control group (all *P* < .001). Regarding hematological parameters, no significant between-group differences were observed in the changes in WBC, RBC, or Hb (all *P* > .05). However, the reduction in PLT was significantly greater in the observation group than in the control group (*P* = .003). These findings describe short-term laboratory differences between the groups and should not be interpreted as evidence of improved cardiovascular prognosis (Table 3).

**Table 3:** Comparison of laboratory parameter changes after treatment between the control group and the observation group.

Variable	Control group (n = 106)	Observation group (n = 109)	Test statistic	*P* value
TC change, mmol/L	−0.51 ± 0.42	−0.90 ± 0.48	*t* = 6.34	<.001
TG change, mmol/L	−0.21 ± 0.29	−0.46 ± 0.35	*t* = 5.71	<.001
LDL-C change, mmol/L	−0.47 ± 0.31	−0.81 ± 0.37	*t* = 7.30	<.001
HDL-C change, mmol/L	0.04 ± 0.11	0.14 ± 0.13	*t* = −6.06	<.001
FIB change, g/L	−0.19 ± 0.28	−0.49 ± 0.34	*t* = 7.05	<.001
D-dimer change, mg/L	−0.07 ± 0.11	−0.18 ± 0.14	*t* = 6.38	<.001
WBC change, ×10^9^/L	−0.33 ± 0.56	−0.39 ± 0.61	*t* = 0.75	.452
RBC change, ×10^12^/L	−0.02 ± 0.09	0.00 ± 0.10	*t* = −1.54	.125
Hb change, g/L	−1.1 ± 6.2	0.3 ± 6.5	*t* = −1.61	.109
PLT change, ×10^9^/L	−3.8 ± 18.5	−11.6 ± 20.2	*t* = 2.96	.003

FIB = fibrinogen, Hb = hemoglobin, HDL-C = high-density lipoprotein cholesterol, LDL-C = low-density lipoprotein cholesterol, PLT = platelet count, RBC = red blood cell count, TC = total cholesterol, TG = triglycerides, WBC = white blood cell count.

### 3.4. Comparison of overall clinical efficacy between the 2 groups

The distribution of overall clinical response differed significantly between the 2 groups (χ^2^ = 9.63, *P* = .008). In the control group, 32 patients (30.2%) were classified as markedly effective, 49 (46.2%) as effective, and 25 (23.6%) as ineffective, whereas in the observation group, 48 patients (44.0%) were classified as markedly effective, 51 (46.8%) as effective, and 10 (9.2%) as ineffective. The overall response rate was significantly higher in the observation group than in the control group (99 [90.8%] vs 81 [76.4%], χ^2^ = 8.19, *P* = .004). This represents an unadjusted association and should not be interpreted as evidence of causal treatment superiority (Table 4).

**Table 4:** Comparison of overall clinical efficacy between the control group and the observation group.

Variable	Control group (n = 106)	Observation group (n = 109)	Test statistic	*P* value
Markedly effective, n (%)	32 (30.2)	48 (44.0)		
Effective, n (%)	49 (46.2)	51 (46.8)		
Ineffective, n (%)	25 (23.6)	10 (9.2)	χ^2^ = 9.63	.008
Overall response rate, n (%)	81 (76.4)	99 (90.8)	χ^2^ = 8.19	.004

### 3.5. Comparison of safety outcomes between the 2 groups

The incidences of gastrointestinal discomfort, allergic reaction, bleeding events, hepatic dysfunction, renal dysfunction, and other treatment-related adverse events were similar between the 2 groups, with no statistically significant differences observed (all *P* > .05). Specifically, gastrointestinal discomfort occurred in 9 patients (8.5%) in the control group and 11 patients (10.1%) in the observation group (*P* = .688); allergic reactions in 3 patients (2.8%) and 2 patients (1.8%; *P* = .627); bleeding events in 6 patients (5.7%) and 4 patients (3.7%; *P* = .494); hepatic dysfunction in 5 patients (4.7%) and 3 patients (2.8%; *P* = .453); renal dysfunction in 2 patients (1.9%) and 2 patients (1.8%; *P* = .979); and other treatment-related adverse events in 4 patients (3.8%) and 5 patients (4.6%; *P* = .767), respectively. In addition, the overall incidence of adverse events did not differ significantly between the control group and the observation group (29 [27.4%] vs 27 [24.8%], χ^2^ = 0.19, *P* = .666). Thus, adjunctive TCM was not associated with a higher observed incidence of documented short-term adverse events in this cohort (Table 5).

**Table 5:** Comparison of adverse events between the control group and the observation group.

Variable	Control group (n = 106)	Observation group (n = 109)	Test statistic	*P* value
Gastrointestinal discomfort, n (%)	9 (8.5)	11 (10.1)	χ^2^ = 0.16	.688
Allergic reaction, n (%)	3 (2.8)	2 (1.8)	χ^2^ = 0.24	.627
Bleeding events, n (%)	6 (5.7)	4 (3.7)	χ^2^ = 0.47	.494
Hepatic dysfunction, n (%)	5 (4.7)	3 (2.8)	χ^2^ = 0.56	.453
Renal dysfunction, n (%)	2 (1.9)	2 (1.8)	χ^2^ = 0.00	.979
Other treatment-related adverse events, n (%)	4 (3.8)	5 (4.6)	χ^2^ = 0.09	.767
Total adverse events, n (%)	29 (27.4)	27 (24.8)	χ^2^ = 0.19	.666

## 4. Discussion

In this retrospective real-world study, patients receiving adjunctive TCM in addition to conventional Western medical therapy had lower rates of residual chest pain, chest tightness, and fatigue; a higher observed clinical response rate; and greater short-term changes in several lipid- and coagulation-related laboratory parameters than patients receiving Western medical therapy alone. The incidence of documented adverse events was similar between groups. However, because treatment allocation was determined by routine clinical practice rather than randomization and the analyses were unadjusted for potential confounding factors, these findings should be interpreted as associations rather than evidence of causal treatment effects. The absence of statistically significant differences in measured baseline characteristics does not exclude residual confounding from unmeasured or incompletely measured factors.

Symptom control remains an important therapeutic target in CAD, in parallel with secondary prevention. Contemporary guidelines for chronic coronary disease and chronic coronary syndromes emphasize not only prevention of cardiovascular events but also reduction of angina burden, improvement in functional status, and optimization of quality of life through comprehensive medical therapy and risk-factor control.^[9]^ Within this context, the lower rates of residual chest pain and chest tightness observed in the integrated-treatment group are consistent with a possible association between adjunctive TCM use and greater short-term symptom relief; however, the present observational study cannot establish that TCM itself caused these differences. This pattern is broadly consistent with recent clinical evidence indicating that integrated Chinese and Western approaches may improve angina-related outcomes in selected populations with stable coronary disease.^[10,11]^

Several mechanisms may plausibly explain the symptomatic advantage observed in the observation group. Coronary symptoms arise from a complex interaction among fixed atherosclerotic burden, endothelial dysfunction, vascular tone abnormalities, inflammation, thrombogenic activity, and microcirculatory impairment. Current guideline frameworks increasingly recognize that outcomes in chronic coronary disease are influenced by more than epicardial stenosis alone, and that metabolic, lipid-related, inflammatory, and thrombotic pathways all contribute to disease activity and symptom persistence.^[12,13]^ The greater reduction in residual fatigue in the integrated-treatment group may also be relevant, because fatigue in coronary populations is often multifactorial and may reflect ongoing ischemic symptoms, impaired exercise tolerance, systemic inflammatory burden, and treatment-related symptom perception rather than a single cardiac variable. In the absence of objective functional testing in the present study, this observation should be interpreted cautiously, but it remains clinically meaningful as part of overall symptomatic recovery.

A notable finding was the greater short-term change in lipid parameters in the observation group, including greater reductions in TC, TG, and LDL-C and a greater increase in HDL-C. Lipid lowering remains a central component of secondary prevention, and LDL-C is an established cardiovascular risk marker. Nevertheless, the present findings reflect short-term biochemical changes and cannot be assumed to translate into reduced cardiovascular events or improved prognosis.^[14,15]^ Recent meta-analytic evidence has suggested that adjunctive Chinese herbal medicine may be associated with additional improvement in lipid profiles in patients with coronary heart disease, although heterogeneity in formulations and study quality remains an important limitation.^[16,17]^ The present data are directionally consistent with these laboratory findings but do not establish prognostic benefit.

The observed differences in coagulation-related markers are also noteworthy. FIB and D-dimer reflect, albeit indirectly, prothrombotic activity, fibrin turnover, and the interaction between inflammation and coagulation. In this study, the observation group showed greater reductions in both FIB and D-dimer. However, these changes represent surrogate laboratory signals and should not be interpreted as direct evidence of reduced thrombotic events or improved prognosis, because the present study did not assess major adverse cardiovascular outcomes, platelet function, or imaging markers of plaque stability. With regard to routine hematological indices, no significant between-group differences were observed in WBC, RBC, or Hb change, whereas PLT change differed significantly between groups. PLT alone is an incomplete surrogate for platelet reactivity or thrombotic risk, and the isolated PLT finding should therefore be interpreted cautiously.^[18–21]^

Another important observation was the comparable safety profile between groups. No significant between-group differences were identified in gastrointestinal discomfort, allergic reactions, bleeding events, hepatic dysfunction, renal dysfunction, other treatment-related adverse events, or total adverse-event incidence. This result is clinically relevant because adjunctive therapies in coronary populations must be judged not only by efficacy but also by compatibility with antiplatelet, lipid-lowering, antihypertensive, and antidiabetic regimens that already constitute standard care. Guideline-based treatment for chronic coronary disease is intensive and frequently involves multidrug combinations, making treatment tolerance and interaction risk central considerations. Recent reviews of Chinese herbal medicine in cardiovascular settings have generally reported acceptable safety profiles when used adjunctively, although the certainty of evidence remains limited by variable reporting standards and heterogeneous intervention protocols.^[22]^ The current findings therefore support, but do not conclusively establish, the short-term safety of integrated therapy in this population.

The present findings are broadly consistent with, but methodologically more limited than, several recent studies of adjunctive TCM in coronary disease. A 2023 real-world study of 690 patients with stable angina reported fewer cardiovascular events with integrated treatment after multivariable and propensity-score analyses. More recently, a 2026 multicenter prospective cohort of 1282 patients with intermediate coronary lesions reported lower long-term major adverse cardiovascular events and major adverse cardiovascular and cerebrovascular events rates with integrated therapy after multivariable, sensitivity, and propensity-score analyses.^[19]^ In contrast to those studies, the present analysis was single-center, retrospective, focused primarily on short-term symptoms and laboratory changes, and did not perform confounder adjustment or evaluate adjudicated cardiovascular events. Thus, our findings should be viewed as complementary descriptive real-world evidence rather than confirmatory evidence of prognostic benefit.

Recent randomized and evidence-synthesis literature also provides useful context. A 2026 multicenter randomized, double-blind trial of Yi’anning pills in stable angina evaluated validated symptom-centered outcomes over 8 weeks, while 2026 systematic reviews and network meta-analyses of TCM or Chinese patent medicines after PCI reported potential improvements in selected cardiac, inflammatory, and cardiovascular outcomes but emphasized heterogeneity and the need for larger high-quality trials. A 2026 mechanistic review further summarized potential effects of TCM on inflammation, oxidative stress, endothelial injury, and lipid metabolism.^[23]^ Compared with these studies, our work reflects individualized syndrome-differentiated TCM in routine practice and therefore offers pragmatic information, but the heterogeneity of treatment and lack of randomized allocation limit direct efficacy comparisons. The main contribution of the current study is therefore to provide hypothesis-generating real-world evidence supporting the need for prospective, adequately controlled studies to determine whether the observed short-term symptomatic and laboratory associations translate into clinically meaningful long-term benefits.

### 4.1. Implications for current practice and contribution to the literature

The new information provided by this study is not sufficient to justify modification of existing CAD treatment standards. Guideline-directed Western medical therapy should remain the foundation of secondary prevention and symptom management. The present data only suggest that, in routine practice at a single center, adjunctive syndrome-differentiated TCM was associated with better short-term symptom status and selected laboratory changes without a detectable increase in documented short-term adverse events. Accordingly, the findings may support further evaluation of adjunctive TCM in appropriately selected patients, but they should not be used to replace established therapies or to infer a reduction in cardiovascular events.

This study adds to the literature by describing a real-world cohort that included patients with different CAD presentations and routine individualized TCM treatment rather than a single fixed formulation. It simultaneously reports symptom burden, lipid- and coagulation-related laboratory changes, a composite clinical-response measure, and documented safety. Its principal contribution is therefore hypothesis generation: it identifies clinically relevant short-term signals that can inform the design and outcome selection of future controlled studies, while also illustrating the limitations of unadjusted retrospective comparisons.

## 5. Limitations

Several limitations should be considered when interpreting the present findings. First, this was a retrospective, non-randomized observational study in which treatment allocation was determined by routine clinical practice. The decision to prescribe adjunctive TCM may have been influenced by disease severity, symptom burden, physician and patient preferences, socioeconomic factors, medication adherence, treatment tolerance, TCM syndrome characteristics, and other factors not fully captured in the medical record. Although measured baseline characteristics did not differ significantly between groups, such apparent similarity does not eliminate confounding by indication or residual confounding. Importantly, no propensity-score or multivariable adjustment was performed; therefore, the observed between-group differences are unadjusted associations and cannot establish causal efficacy.

Second, several outcomes, particularly symptom status and the overall clinical-response classification, were retrospectively abstracted from clinical documentation and may be susceptible to information and ascertainment bias. Objective laboratory variables reduce, but do not remove, this concern. Third, the study assessed short-term symptoms and surrogate laboratory markers rather than adjudicated major adverse cardiovascular outcomes. Changes in lipid profiles, FIB, D-dimer, or PLT cannot be interpreted as evidence of reduced myocardial infarction, cardiovascular mortality, recurrent ischemic events, or improved long-term prognosis.

Fourth, although follow-up timing was assessed within the predefined posttreatment window and no systematic between-group difference was identified, residual variation in measurement timing is inherent to retrospective real-world data. Fifth, TCM therapy was individualized according to syndrome differentiation; heterogeneity in formulation, dose, treatment duration, and subsequent modification reduces intervention standardization and reproducibility.

Finally, this was a single-center study with a moderate sample size and limited follow-up. These features restrict generalizability to other institutions, regions, TCM prescribing patterns, and CAD populations. The number of outcomes assessed also raises the possibility of chance findings due to multiple comparisons. Taken together, these limitations require cautious interpretation of the observed associations.

## 6. Knowledge gaps

Important knowledge gaps remain. It is uncertain which patient phenotypes or TCM syndrome subgroups, if any, are most likely to benefit from adjunctive therapy; which specific formulations, components, doses, and treatment durations are optimal; and whether observed short-term symptom or biomarker changes translate into durable improvements in validated health-status measures or cardiovascular outcomes. The independent contribution of TCM is also difficult to separate from differences in adherence, physician behavior, background pharmacotherapy, lifestyle management, and other unmeasured factors in routine care. In addition, standardized definitions of TCM exposure, harmonized outcome sets, rigorous adverse-event surveillance, and clinically meaningful thresholds for symptom improvement are lacking across much of the literature.

## 7. Future directions

Future research should prioritize prospective multicenter studies with prespecified treatment and assessment schedules, standardized characterization of TCM formulations and syndrome differentiation, and validated patient-centered outcomes such as the Seattle Angina Questionnaire. Randomized controlled trials are preferred when feasible. High-quality observational studies should use rigorous methods to address treatment-selection bias, including comprehensive covariate measurement, propensity-score or other causal-inference approaches, sensitivity analyses for unmeasured confounding, and transparent reporting of missing data. Longer follow-up with independently adjudicated major adverse cardiovascular events, hospitalization, quality of life, functional capacity, medication adherence, drug-herb interactions, and safety outcomes is necessary to determine whether any short-term associations have clinical relevance sufficient to influence treatment decisions.

## 8. Conclusion

In this retrospective, real-world cohort of patients with CAD, adjunctive TCM was associated with greater short-term improvement in selected symptoms, lipid- and coagulation-related laboratory parameters, and observed clinical response compared with conventional Western medical therapy alone. No significant difference in documented short-term adverse events was observed between groups. However, because treatment allocation was nonrandom and the analyses were not adjusted for potential confounding factors, causal superiority cannot be established. In addition, changes in laboratory biomarkers should not be interpreted as evidence of improved cardiovascular prognosis. These findings should therefore be considered exploratory and require confirmation in prospective, appropriately controlled studies.

## Author contributions

**Conceptualization:** Ziyi Chai, Huaying Huo.

**Data curation:** Ziyi Chai, Huaying Huo.

**Formal analysis:** Ziyi Chai, Huaying Huo.

**Funding acquisition:** Huaying Huo.

**Investigation:** Huaying Huo.

**Writing – original draft:** Huaying Huo.

**Writing – review & editing:** Huaying Huo.
